# Digitalization of presence events in the COVID-19 pandemia – the lecturers’ perspective

**DOI:** 10.3205/zma001426

**Published:** 2021-01-28

**Authors:** Marc Gottschalk, Katrin Werwick, Christian Albert, Soenke Weinert, Alexander Schmeißer, Philipp Stieger, Ruediger C. Braun-Dullaeus

**Affiliations:** 1 Otto-von-Guericke-Universität Magdeburg, Universitätsklinik für Kardiologie und Angiologie, Magdeburg, Germany; 2 Otto-von-Guericke-Universität Magdeburg, Medizinische Fakultät, Studiendekanat, Magdeburg, Germany; 3 Otto-von-Guericke-Universität Magdeburg, Universitätsklinik für Kardiologie und Angiologie, AG Lehre in der Kardiologie, Magdeburg, Germany; 4 MVZ Potsdam, Diaverum Renal Services, Potsdam, Germany

**Keywords:** online courses, teaching, COVID-19, pandemic

## Abstract

**Objective: **Due to the COVID-19 pandemic a large part of attendance in medical education became impossible for reasons of disease control. Teachers had to switch to online courses at short notice. The associated developmental push of digital teaching methods, such as online teaching, has anticipated changes, some of which are tantamount to establishment. This study examines the experiences and effects of these changes from the teachers’ perspective.

**Methods:** We conducted ten guideline-based anonymized e-mail interviews with lecturers of the Medical Faculty of the Otto-von-Guericke University Magdeburg. Questions were asked on the subject areas of advantages and disadvantages, teaching experience and the future of digital teaching. The qualitative evaluation was based on Mayring.

**Results: **The assessment of the digitization of face-to-face courses could be described by the inductively formed categories “social aspects”, “methodological aspects”, “institutional aspects”, “technical aspects” and “temporal-spatial aspects”. These revealed in particular concerns about the lack of personal exchange, temporal-spatial advantages, technical barriers and disagreement about the future role of digital teaching.

**Conclusion:** In the context of the COVID-19 pandemic, face-to-face courses were replaced by online teaching, which is currently an accepted part of the curriculum. The results show, that teachers were able to implement the comprehensive ad-hoc digitization of theoretical courses well, although previously known problem areas were aggravated. Furthermore, a fundamental examination of the future role of digitized courses in medical education must take place.

## Zusammenfassung

**Zielsetzung: **Durch die COVID-19-Pandemie wurde ein Großteil medizinischer Präsenzveranstaltungen aus Gründen des Seuchenschutzes unmöglich. Lehrende mussten kurzfristig auf online-Lehrveranstaltungen ausweichen. Der damit verbundene Entwicklungsschub digitaler Lehrmethoden, wie z.B. online-Unterricht, hat Veränderungen vorweggenommen, die teilweise einer Etablierung gleichkommen. In dieser Studie werden Erfahrungen und Effekte im Zuge dieser Veränderungen aus Sicht der Lehrenden untersucht.

**Methodik: **Wir führten zehn leitfadengestützte anonymisierte E-Mail-Interviews mit Lehrenden der Medizinischen Fakultät der Otto-von-Guericke-Universität Magdeburg durch. Es wurden Fragen zu den Themenkomplexen Vor- und Nachteile, Lehrerleben und Zukunft digitaler Lehre gestellt. Die Auswertung erfolgte qualitativ nach Mayring.

**Ergebnisse:** Die Einschätzungen zur Digitalisierung von Präsenzveranstaltungen ließen sich durch die induktiv gebildeten Kategorien „soziale Aspekte“, „methodische Aspekte“, „institutionelle Aspekte“, „technische Aspekte“ sowie „zeitlich-räumliche Aspekte“ beschreiben. Es zeigten sich in diesen besonders die Sorge um mangelnden persönlichen Austausch, zeitlich-räumliche Vorteile, technische Barrieren und Uneinigkeit über die zukünftige Rolle digitaler Lehre.

**Schlussfolgerung: **Im Rahmen der COVID-19-Pandemie wurden Präsenzveranstaltungen durch online-Unterricht ersetzt, dieser ist aktuell akzeptierter Bestandteil des Studiums. Die Ergebnisse zeigen, dass Lehrende die umfassende ad-hock-Digitalisierung theoretischer Lehrveranstaltungen gut umsetzen konnten, wenngleich vorbekannte Problemfelder aggravierten. Weiterhin muss eine grundlegende Auseinandersetzung mit der zukünftigen Rolle digitalisierter Lehrveranstaltungen in der medizinischen Ausbildung stattfinden.

## 1. Introduction

Due to the COVID-19 pandemic, many face-to-face courses became impossible for reasons of disease control [1]. Solutions had to be developed within a short time at the Magdeburg campus, which resulted in a previously unexpected offer of digitalized courses. After the completion of these classes, changes in medical education became apparent. The discrepancy between recently dominant traditional courses and now established online courses exists due to the hitherto low presence of digital courses in the German-speaking world. Until it became necessary due to the pandemic regulations, the low level of development was justified by a lack of infrastructure, limited time resources, and a lack of institutional support [2], [3]. Little is known about the teachers’ experience of the transition to digital formats. The aim of this paper is to describe the teachers’ experiences with this new situation and to assess its effects.

## 2. Methodology

After the end of the 2020 summer semester, we conducted ten anonymous, guideline-based expert interviews [4], [5] with lecturers from the Medical Faculty of the Otto-von-Guericke University of Magdeburg. These were conducted as e-mail interviews [6]. The lecturers answered in writing to a ready-made guide consisting of open questions on the subject areas of advantages and disadvantages, teaching experience and the future of digital courses. The material was analyzed qualitatively according to Mayring [7] and transferred into an inductively formed category system. To improve intersubjective comprehensibility, we used a research workshop [8], [9] consisting of the authors listed above to interpret the material (see attachment 1 ).

## 3. Results

Five categories were inductively formed from the statements made by the respondents (see table 1 (Tab. 1)). With regard to “temporal-spatial aspects”, the teachers described positive experiences regarding independence of place, time and the possibility of individualizing the learning environment. On the subject of “technical aspects”, they expressed enthusiasm for the use of new technologies, but complained about limitations of hardware and software. Problems were seen by many teachers concerning the lack of direct, personal interaction, which they found unsettling and frustrating. At the same time, however, they saw potential in location-independent exchange. Regarding “methodological aspects”, the teachers described theoretical courses as suitable for digitization. Internships and bedside teaching were viewed critically. When looking at “institutional aspects”, different perspectives were noted in addition to the demand for more support: While some lecturers saw digital courses as a supplement to a curriculum that continues to be primarily presence-based, others anticipated lasting changes in medical faculties with increased digital teaching, but also expressed fears that this could come at the expense of academic exchange (see table 1 (Tab. 1)).

## 4. Discussion

With the restrictions imposed by epidemic control regulations, new approaches had to be taken in teaching. One solution was the short-term establishment of online courses. From the teachers' point of view, this was a development that allowed traditional academic ideas to be reassessed. Teachers experienced the practicability and flexibility of digitized courses as advantageous, but at the same time they criticized the lack of personal exchange and technical barriers.

The advantages in terms of time and space can be connected to Ruiz [10] and became particularly clear through the comprehensive implementation. Technical problems and a lack of support have already been identified as barriers to the further development of digital teaching formats [3], but they have been aggravated by the increasing demand during the pandemic. While teaching formats such as lectures were perceived as easily digitizable, there were concerns about the lack of interactivity in the implementation of seminars and practical courses. However, the large number of published pilot projects [11], [12], which could provide pointers for optimization, were only considered by a few teachers. Regarding institutional challenges and the future role of online courses, initiatives to coordinate the digitization of medical education have already been called for in the past [13], but currently there is still disagreement about the future use of digital courses. In order to effectively improve medical education, it is therefore necessary to explore the perspective of teachers and students beyond the scope of this study. The findings of our study can serve as a basis for this.

One of the limitations of this study is the small number of interviews, which limits the generalization of the results [8]. The methodology of e-mail interviews was chosen to reduce contact, and a loss of information [6] was accepted to protect against the epidemic. An already validated guideline could not be used due to the novelty of the question.

## 5. Conclusion

In the context of the COVID-19 pandemic classroom teaching was replaced by online teaching, which is currently an accepted part of the curriculum. The results show that teachers were able to implement the comprehensive ad-hoc digitization of theoretical courses. Although known problem areas were aggravated during the crisis, the advantages are undeniable. Ultimately, a more in-depth examination of the future role of digital courses in medical education is called for.

## Contributions of the authors

All authors have contributed according to the criteria of the International Committee of Medical Journal Editors [http://www.icmje.org/recommendations/browse/roles-and-responsibilities/defining-the-role-of-authors-and-contributors.html] made substantial contributions to the conception, design and analysis of the study. Research idea RCBD and PS. PS and KW designed the questionnaire. MG and PS acquired and evaluated the interview data. MG and CA prepared the first draft of the manuscript. All authors participated in the interpretation of the data in a research workshop and in the critical revision of the publication and made substantial contributions to the content. All authors accepted responsibility for the entire content and gave their final approval of the submitted manuscript version.

## Primary data

The data sets generated and/or analyzed during the current study are available upon reasoned request from the authors.

## Acknowledgement

We would like to thank all participating teachers and all participating students.

## Competing interests

The authors declare that they have no competing interests. 

## Supplementary Material

Supplemental material

## Figures and Tables

**Table 1:**
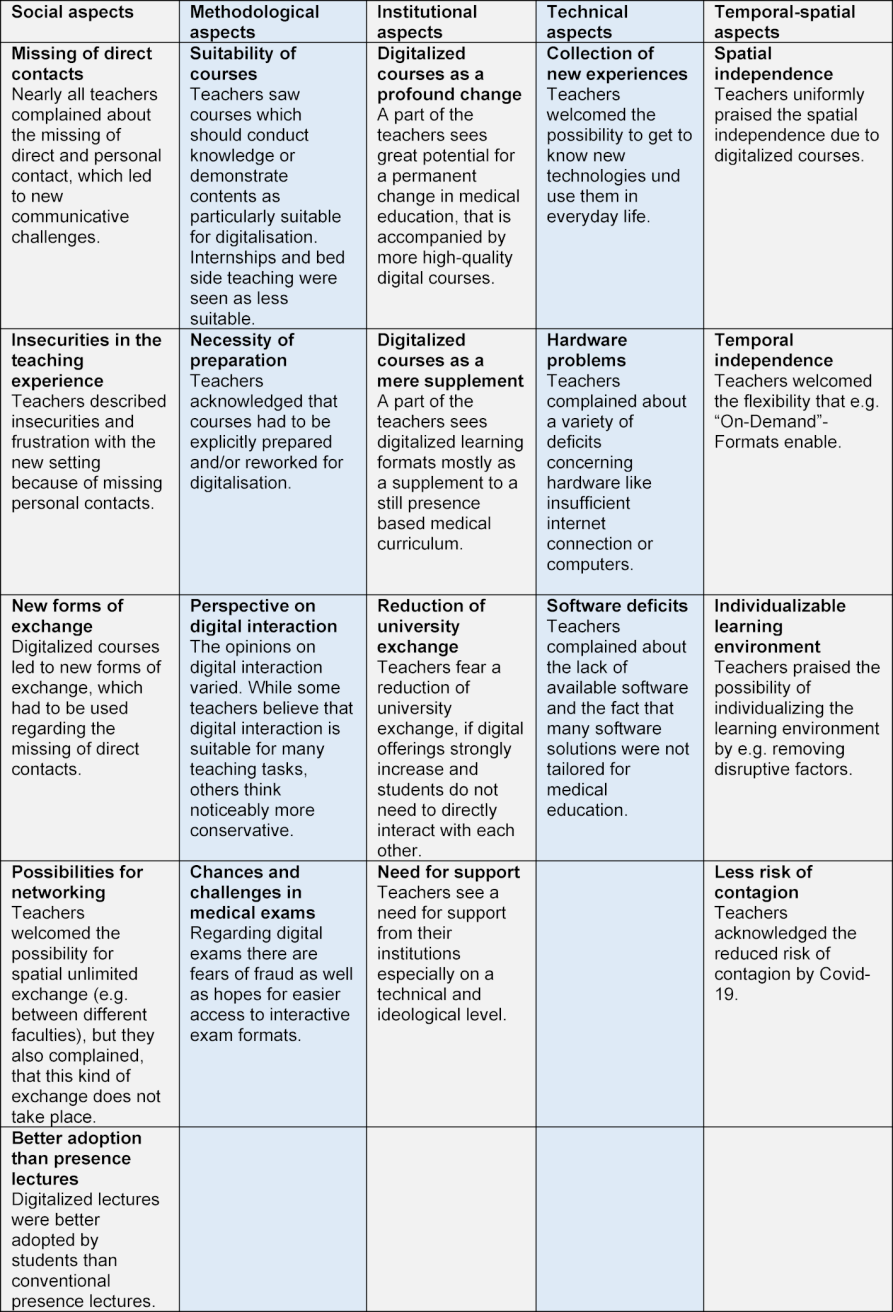
Results of the analysis of the e-mail interviews by qualitative content analysis according to Mayring.
