# Supplementary material for: Digitalization of presence events in the COVID-19 pandemia – the lecturers’ perspective
Source: GMS J Med Educ. 2021 Jan 28;38(1):Doc30. doi: 10.3205/zma001426 (PMC7899118; doi:10.3205/zma001426)
Supplement: Supplemental material [file JME-38-1-30-s-001.pdf]

Attachment 1: Supplemental material

Notes on methodology

In this study, guideline-based expert interviews were conducted via e-mail. Guideline-based interviews [5] are a standard procedure in qualitative social research. First, a guideline is created, which is divided into several sections and contains questions on various topics. These are usually open-ended, allowing the interviewee as much freedom as possible to answer them [8]. In our work, we asked questions on advantages and disadvantages, teaching experience and the future of digitalized courses. Expert interviews with persons who have special knowledge, e.g. about the organization and implementation of university courses, are usually conducted as guided interviews [4]. E-mail interviews are still relatively new in qualitative research, but are sufficiently described in the literature [6]. In the context of the Covid-19 pandemic, they are a good tool to enable research without risk – even in times of contact restriction. In e-mail interviews the respondent receives the guide in written form, which he or she also answers in writing and returns digitally to the interviewer. According to Hunt [6], this has the disadvantage, that it can lead to a loss of information due to the lack of gestures and facial expressions, it is impersonal and a loss of focus due to poor steering is possible. Advantages are, among other things, the already mentioned contactless execution, the possibility of the interviewee to reflect in the writing process as well as the immediate writing. This was followed by a content analysis according to Mayring [7], which consisted of a sequence of generalization, reduction, and summarization, which led to the inductive formation of a category system (see table 1). A research workshop [8, 9] consisting of the above-mentioned authors was used to improve intersubjective traceability. In research workshops qualitative material was presented and jointly analysed. The use of several interpreters enables a higher quality of the categories obtained.

Attachment 1 to: Gottschalk M, Werwick K, Albert C, Weinert S, Schmeißer A, Stieger P, Braun-Dullaeus R. Digitalization of presence events in the COVID-19 pandemia – the lecturers’ perspective. GMS J Med Educ. 2021;38(1):Doc30. DOI: 10.3205/zma001426
